# The Clean-Label-Friendly Solution: Stabilizing Milk-Based Cocoa Beverages with Citrus Fiber

**DOI:** 10.1021/acsomega.5c01904

**Published:** 2025-07-17

**Authors:** Saniye Akyıl Öztürk, Deniz Damla Altan Kamer, Ali Can Karaca, Omer Said Toker

**Affiliations:** † Chemical and Metallurgical Engineering Faculty, Food Engineering Department, 52999 Yildiz Technical University, 34220 Istanbul, Turkey; ‡ Azelis Application & Training Center, 34774 Istanbul, Turkey; § Department of Food Engineering, Faculty of Agriculture, 162334 Tekirdag Namik Kemal University, 59030 Tekirdag, Turkey; ∥ Department of Computer Engineering, Yıldız Technical University, 34220 Istanbul, Turkey

## Abstract

The increasing demand for clean label products has driven the development of natural and sustainable stabilizers in the food industry. This study investigates the potential of citrus fiber as a clean-label-friendly ingredient to improve the stability of cocoa-based dairy beverages. A Central Composite Rotatable Design (CCRD) was employed to evaluate the effects of homogenization pressure (100–300 bar) and citrus fiber concentration (0.10–0.40%) on the water-holding capacity (WHC), sedimentation, creaming index, pH, viscosity, and torque. Analytical methods including zeta potential, FTIR, particle size distribution, microscopy, and SEM–EDS were utilized to assess the structural properties and stability of the formulations. The results demonstrated significant improvements under optimal conditions that citrus fiber significantly enhanced WHC (95.95%), minimized sedimentation (0%) with creaming index (0%), and improved viscosity (197.22 mPa·s), contributing to a homogeneous dispersion of cocoa particles. Homogenization pressure and citrus fiber concentration exhibited synergistic effects, optimizing the physical stability of the beverage. Zeta potential (−27.90 mV) and particle size (630.53 nm) distribution analysis indicated improved electrostatic stability and reduced particle aggregation, while FTIR revealed interactions between citrus fibers and cocoa components. Microscopic analysis, molecular docking, and SEM–EDS further confirmed the structural integration of citrus fiber and proteins, reducing sedimentation and creaming index phenomena. This study underscores the functional advantages of citrus fibers as a natural stabilizer, offering a clean, label-friendly solution for the dairy beverage industry. The findings provide valuable insights for product formulation and process optimization, paving the way for the development of stable, high-quality cocoa-based dairy beverages that align with sustainable ingredients.

## Graphical abstract

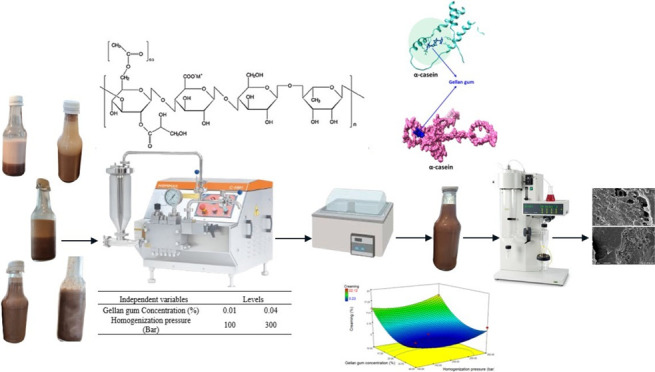

## 1 Introduction

Recent developments in food science and consumer behavior have led to increased interest in formulating “clean label-friendly” products, which are characterized by the use of minimally processed, familiar, and nature-derived ingredients. This demand has prompted food manufacturers to reconsider the use of traditionally processed stabilizers such as hydroxypropyl methylcellulose (HPMC), xanthan gum, and emulsifiers which, although functionally effective, are often perceived as undesirable due to complex processing.^1,2^ In this context, naturally derived ingredients, such as plant-based proteins, native starches, and dietary fibers, have gained attention for their multifunctional properties in food stabilization. Among these, citrus fiber obtained as a byproduct of the juice industry presents a promising alternative due to its ability to improve water-holding capacity, contribute to viscosity, and enhance physical stability without the need for extensive chemical modification. Its use supports both clean-label-friendly formulation and circular economy principles by valorizing food-processing side-streams.^3,4^ However, there is limited information available on the stabilization of milk-based cocoa beverages by citrus fiber.

The stabilization of milk-based cocoa beverages poses significant formulation challenges, particularly under high-temperature processing and extended shelf life requirements. Conventional hydrocolloids and gums, including pectin, carrageenan, modified starches, and HPMC, are commonly employed to prevent phase separation, sedimentation, and creaming.^5^ However, these ingredients are increasingly being scrutinized by consumers seeking transparency.

Stabilizers are important in developing stable colloidal textures, such as emulsions, by retarding the coalescence or flocculation of emulsion droplets. It has been shown that several compounds might be used to stabilize emulsions as food colloids. Commonly they are powders, such as proteins, starch, or dietary fibers. In recent years, the food industry’s attention has been drawn to the potential of citrus fibers, where they have been shown to possess the ability to stabilize emulsions. In addition, possibilities to produce a wide range of stable emulsified products have been demonstrated.^5,6^

Citrus fiber has shown potential to interact with milk proteins to form stable colloidal systems, thereby improving physical stability in emulsified dairy beverages.^7^ Its ability to act as a water binder, fat mimetic, and calcium chelator contributes to texture enhancement and reduction of phase instability, such as sedimentation or fat separation.^8^ Despite these advantages, studies on the application of citrus fiber as a primary stabilizer in milk-based cocoa beverages remain limited.

The present study investigates the use of citrus fiber as a nature-derived stabilizing agent in milk-based cocoa beverages, evaluating its effects on the physicochemical stability parameters. The findings suggest that citrus fiber may offer a viable alternative to synthetic or highly processed stabilizers, contributing to the development of clean-label-friendly and nutrition-forward formulation strategies without compromising product performance.

## 2 Materials and Methods

### 2.1 Materials

Pasteurized milk (3% fat, 3% protein) and crystal sugar were obtained from a local market. Citrus fiber powder (NUTRAVA Citrus Fiber Zest) (40% insoluble fiber, 45% soluble fiber, 5% protein, and 12% moisture) was kindly donated by CP Kelco (Brazil). Citrus fiber is primarily obtained from the byproducts of citrus fruit processing, especially from the peels and pulp of fruits such as orange, lime, and lemon. Alkalized cocoa powder (10–12% fat, black-brown color, strongly alkalized with a pH of 8.80) was supplied by Altinmarka (Turkey), dipotassium phosphate was supplied by Budenheim (Germany), and potassium sorbate was supplied by Nantong (China). All chemicals used throughout the study were purchased from Sigma-Aldrich Chemie (Steinheim, Germany).

### 2.2 Production of Milk-Based Cocoa Beverage and Experimental Design

Sugar (6.00%), alkalized cocoa powder (1.00%), dipotassium phosphate (1.00%), potassium sorbate (0.05%), and citrus fiber were mixed in dry form. Dairy industry makes a dry mixture and adds this dry mixture to milk. The dry phase was added to the mixed pasteurized milk, and all components were mixed at 450 rpm in Thermomix TM6 (Thermomix TM6, Vorwerk, Germany). The system was heated to 75 °C and immediately homogenized (Hommak C-HM1, zmir, Turkey) at the pressure norms (between 58 and 341 bar) specified in the CCRD experimental design. After homogenization, the sample was kept in a Thermomix TM6 at 95 °C/4 min. The system was cooled very quickly in the water bath (Nukleon NBS-30) to 25 °C and the sample was filled into glass vials with plastic caps.^9^

This study aimed to investigate the effects of variables (citrus fiber and homogenization pressure) on the physical stability of milk-based cocoa beverages. Key stability parameters assessed included water-holding capacity (WHC), which indicates the system’s ability to retain water and resist phase separation; sedimentation, which measures the extent of insoluble component settling over time; and the creaming index, reflecting fat destabilization and separation. These parameters were evaluated to optimize formulation stability and assess the suitability of citrus fiber as a clean-label-friendly stabilizer in cocoa milk beverages under dairy-industry-relevant processing conditions. Following the optimization study, the formulated milk-based cocoa beverage was compared to the product without hydrocolloid milk-based cocoa beverage with respect to the optimized citrus fiber concentration and homogenization pressure level. Microscopic and SEM images of the commercial cocoa beverage (1.50% fat, 3% protein, and 1% cocoa powder) were compared with the milk-based cocoa beverage formulated under the optimized conditions and without hydrocolloid milk-based cocoa beverage.

The experimental design matrix and all data analyses were performed using Design-Expert (version 7.0, Stat-Ease Inc., Minneapolis, MN, USA). The Response Surface Methodology (RSM) Central Composite Rotatable Design (CCRD) option was used to examine the effects of citrus fiber concentration and homogenization pressure, two independent variables, on milk-based cocoa beverage properties. The Central Composite Rotatable Design (CCRD) was chosen due to its ability to efficiently explore the effects of multiple variables and their interactions while minimizing the number of experiments required. This design is well-suited for optimization studies, where both linear and quadratic effects need to be evaluated. Experimental design consists of 11 experimental steps, and the effects of independent variables on WHC, sedimentation, creaming index, pH, and viscosity values were investigated. The experimental data were fitted to quadratic models to obtain the regression coefficients. All responses were evaluated for statistical significance of the factors by the ANOVA (Tukey) test. The level range of citrus fiber concentration (0.10–0.40%) and homogenization pressure (100–300 bar) was determined using CCRD experimental design, depending on the industrial applications. The selected range of citrus fiber concentration and homogenization pressure was determined by preliminary experiments. All the responses were evaluated in terms of statistical significance of factors and *R* ^2^ values. For each response variable, a second-order polynomial model equation was defined. Finally, validation studies were carried out under the optimum conditions obtained, and L*, a*, and b* color values, zeta potential value, particle size, FTIR, and microscope images were measured in the samples under these conditions. Optimization was conducted by minimizing sedimentation and creaming index and maximizing WHC considering the desirability value.

### 2.3 Water-Holding Capacity (%)

The water-holding capacity of milk-based cocoa beverages was determined by using centrifugation (Nuve, NF 400, Turkey) of a 50 mL sample at 4000 rpm for 10 min at room temperature. The released serum was measured as a volume (mL).^10^

WHC%=[1−[releasedserum(supernatant)/originalgel]×100

### 2.4 Sedimentation (%)

The prepared milk-based cocoa beverages taken into glass bottles closed with a plastic cap were stored at 4 ± 1 °C for 21 days. The sedimentation value (%) was expressed as the ratio of the height of the sediment at the bottom to the height of the milk-based cocoa beverages.^11^

$$sedimentation\;\%=\left(H_{S}/H_{T}\right)\times 100$$

where *H*_S_ is the height of the sediment and *H*_T_ is the total height of the milk-based cocoa beverages.

### 2.5 Creaming Index (%)

The prepared milk-based cocoa beverages were transferred to a glass bottle closed with a plastic cap and stored at 4 ± 1 °C for 21 days. During this time, the emulsion samples were separated into two layers. These layers were distinguished by visual appearance.^12^ The creaming index (CI) of emulsions is calculated by the following equation:

$$CI\;\%=\left(H_{C}/H_{T}\right)\times 100$$

where *H*_C_ is the height of the cream layer (top) and *H*_T_ is the total height of the milk-based cocoa beverages.

### 2.6 pH

pH values of last products (milk-based cocoa samples) were measured at 25 °C 1 day after production.

### 2.7 Viscosity (mPa·s)

Viscosity measurements were carried out with a viscometer (Lamy B-One Plus, France) at a controlled temperature of 25 °C with a chosen speed of 100 rpm. Measurements were taken 2 min after the spindle (Spindle no. R-2) was immersed in each sample and to allow thermal equilibrium in the samples. Each measurement was triplicated on the same sample.

### 2.8 Color

The color was determined with a digital colorimeter (Konica Minolta, Chroma meter CR-410, Japan). L* indicates the lightness, a* and −a*, redness and greenness, and b* and −b*, yellowness, and blueness, respectively, according to the CIE L*a*b* color space.

### 2.9 Fourier Transform Infrared (FTIR) Spectroscopy

FTIR spectra of optimum point milk-based cocoa beverages were recorded by a FTIR spectrophotometer (Thermo Nicolet, AVATAR-370 FTIR, USA) at a 4000–400 cm^–1^ wavenumber range.^13^ The FTIR spectrophotometer was calibrated using standard reference materials prior to each measurement to ensure the accuracy and reliability of spectra collected.

### 2.10 Zeta Potential Value and Particle Size

The optimum point milk-based cocoa sample was diluted 100 times with phosphate buffer (5 mM). ζ-Potential of the diluted sample was measured using Malvern Mastersizer Nano ZS90 (Malvern Instrument Co. Ltd., UK) at 25 °C, and measurements were performed in triplicate. Droplet size distribution was measured by a Mastersizer 2000 instrument (Malvern Instruments Co., LTD, UK). Regarding particle size measurement, 1 mL of sample was diluted 1000 times with distilled water. The refractive indices were set at 1.33 for the continuous phase and 1.46 for the dispersed phase. The mean diameter value (*d*_4,3_) was calculated by

$$d_{4,3}=n_{i}{d_{i}}^{4}/\sum n_{i}{d_{i}}^{3}$$

$$specific\;surface\;area=\frac{6}{\rho \times d_{3,2}}$$

where *n*_*i*_, *d*_3,2_, and ρ are the number of droplets of diameter *d*_*i*_, surface mean diameter, and density of sample, respectively. The measurements were performed after the milk-based cocoa beverage preparation, and results were obtained from an average of three readings.

### 2.11 Microscopic Analysis

The microstructure of milk-based cocoa beverage produced with citrus fiber at the optimum point was examined using a microscope (Zeiss EVO LS 10, Germany) and was magnified 10 and 40 times under the microscope. To explore the properties of the images, each image was analyzed using a combination of Gray Level Co-occurrence Matrix (GLCM) and a local window analysis procedure, implemented in MATLAB software. Specifically, the analysis began with the extraction of nonoverlapping *W* × *W* patches from the images of the samples, where *W* represents the size of the local window. For each patch, statistical and textural properties were calculated to assess the structural characteristics. These properties included entropy, variance, and homogeneity, derived by using GLCM. Entropy was computed as a measure of randomness or disorder within each patch, capturing the complexity of the image texture. Variance was calculated to quantify the spread of pixel intensity values around their mean, reflecting the degree of contrast within the patches. Homogeneity, derived from GLCM, measured the uniformity of the intensity distribution, indicating the consistency of the texture. The method systematically varied window W to evaluate its impact on the calculated features, ensuring a robust comparison across different samples. By leveraging MATLAB’s computational capabilities to consider a range of patch sizes and extract multiple features, the approach provided a comprehensive characterization of the sample’s structural and textural properties.

### 2.12 Scanning Electron Microscopy (SEM) and Energy-Dispersive Spectroscopy Analysis (EDS)

The microstructures of milk-based cocoa beverage samples produced with citrus fiber at the optimum point and without hydrocolloid for comparison and supplied from the market samples were analyzed using SEM–EDS (JEOL JIB-4601 MultiBeam FIB-SEM system, Japonya). The samples were frozen at −20 °C and freeze-dried for 48 h in a vacuum freeze-dryer (Liyolife, FD5CT, Manisa, Turkey). Scanning electron microscopy (SEM) and energy-dispersive spectroscopy analysis (EDS) was carried out to reveal the microscopic features and chemical mapping. Evaluation metrics extracted from nonoverlapping patches on SEM images were also studied. The method was explained in detail in the microscope images, Section 2.11.

### 2.13 Molecular Docking

The interactions between major proteins in cow milk and citrus fibers were explored through molecular simulations of protein–ligand complexes. Docking simulations were conducted using AutoDock Vina (Pyrex; https://pyrx.sourceforge.io), Discovery Studio (https://www.3ds.com/products/biovia/discovery-studio), and UCSF Chimera (University of California, San Francisco, CA, USA) for virtual screening. The 3D structures of α-lactalbumin (α-LA) and β-lactoglobulin (β-LG) (PDB IDs: 6IP9 and 3NPO) were obtained from the RCSB Protein Data Bank (http://www.rcsb.org), with water molecules and nonessential ligands removed.^14,15^ The I-TASSER server was employed to generate the 3D models for αs1-casein (αs1-CA) and β-casein (β-CA).^16^ The chosen proteins α-lactalbumin, β-lactoglobulin, αs1-casein, and β-casein were selected due to their significance as the primary structural and functional constituents of milk, which directly affect colloidal stability. These proteins serve as primary candidates for citrus fiber interactions, rendering them crucial for investigating the mechanisms behind the reported increases in physical stability. Due to the composition of citrus fiber, which contains approximately 40% cellulose, cellulose was selected as the ligand for molecular docking studies. The molecular structure of cellulose was retrieved from PubChem and drawn using ChemDraw. Proteins Plus (DoG Site Scorer) (https://proteins.plus/) and the PLIP tool (https://plip-tool.biotec.tu-dresden.de/plip-web/plip/index) generated the corresponding 2D and 3D interaction diagrams. In the molecular docking stage, it was assumed that other ingredients in the environment did not affect the interaction, and it was aimed to examine only the interaction between milk proteins and citrus fiber.

### 2.14 Storage Analysis

Samples produced under optimum conditions were subjected to storage analysis over a period of 9 months. During the storage period, parameters such as the water-holding capacity (WHC), sedimentation, creaming, pH, and viscosity were measured at regular intervals. WHC, sedimentation, and creaming index rates were monitored to evaluate long-term stability and the preservation of product homogeneity. Viscosity measurements were performed to observe potential changes in product consistency due to interactions between components or structural changes over time. pH values were measured to determine the maintenance of the product’s acidic properties. All analyses were conducted at specified time points, and the results were statistically evaluated.

### 2.15 Sensory Evaluation

The milk-based cocoa beverage produced at the optimum point and milk-based cocoa beverage containing no hydrocolloid were kept at 4 ± 1 °C for 24 h. Sensory evaluation was carried out by a panel of 10 trained chocolate technology experts, aged 20 to 32 years (4 men, 6 women). The panelists were selected from master’s degree students taking a “sensory analysis” course at Yildiz Technical University, ensuring they possessed the necessary expertise and experience for the sensory assessment process. A 5-point hedonic scale test was used for the sensory analysis, which consisted of smoothness, mouth feeling (texture and creaminess), homogeneity, flavor, and overall acceptability (1 = very bad, 2 = bad, 3 = middle, 4 = good and 5 = excellent).^17^

### 2.16 Statistical Analysis

The results were given as the average value ± standard deviation. The significance of the independent factor was determined using the ANOVA test. The differences among the samples were determined using the Tukey test. ANOVA was used to determine the statistical significance of the factors on the response variables. Tukey’s test was applied for pairwise comparisons among the experimental conditions. All of these analyses were conducted by the SPSS program at a significance level of 0.05.

## 3 Results and Discussion

Some quality characteristics of milk-based cocoa beverages manufactured with different conditions according to the experimental design and the findings related to model can be seen in Table 1. Additionally, images of milk-based cocoa beverages produced at these experimental points are shown in Figure 1.

**Table 1:** Response Values Obtained with the Run Points of the Created CCRD Experimental Design^a^

run	citrus fiber concentration (%)	homogenization pressure (bar)	WHC (%)	sedimentation (%)	creaming index (%)	pH	viscosity (mPa·s)	torque (mN·m)
1	0.100	300	82.90 ± 0.71	3.96 ± 0.29	29.31 ± 0.11	6.65 ± 0.03	94.36 ± 0.79	0.424 ± 0.011
2	0.462	200	97.60 ± 0.57	0.00 ± 0.00	0.00 ± 0.00	6.64 ± 0.01	225.80 ± 1.22	0.941 ± 0.009
3	0.250	200	86.05 ± 0.35	0.00 ± 0.00	0.00 ± 0.00	6.64 ± 0.05	138.30 ± 0.55	0.621 ± 0.023
4	0.250	341.4	90.72 ± 0.68	0.00 ± 0.00	0.00 ± 0.00	6.64 ± 0.01	157.80 ± 0.81	0.709 ± 0.005
5	0.400	300	95.35 ± 0.49	0.75 ± 0.12	2.46 ± 0.22	6.63 ± 0.02	201.60 ± 1.08	0.905 ± 0.027
6	0.038	200	75.65 ± 0.49	17.50 ± 1.18	59.42 ± 0.82	6.64 ± 0.04	76.91 ± 0.24	0.346 ± 0.012
7	0.100	100	77.00 ± 0.85	17.96 ± 0.53	59.15 ± 2.07	6.65 ± 0.02	77.99 ± 0.30	0.350 ± 0.006
8	0.250	58.5	79.25 ± 0.35	6.58 ± 0.82	31.27 ± 0.71	6.64 ± 0.01	124.50 ± 0.43	0.559 ± 0.024
9	0.250	200	85.55 ± 0.21	0.00 ± 0.00	2.46 ± 0.22	6.63 ± 0.03	126.40 ± 0.46	0.568 ± 0.018
10	0.250	200	85.34 ± 0.37	0.00 ± 0.00	0.69 ± 0.11	6.64 ± 0.04	134.20 ± 0.58	0.603 ± 0.025
11	0.400	100	92.36 ± 0.23	0.00 ± 0.00	0.00 ± 0.00	6.63 ± 0.01	161.10 ± 0.66	0.724 ± 0.014
model			linear	quadratic	quadratic		linear	
*p* value			0.0473*	0.0002*	0.0002*		0.0001*	
*R* ^2^			0.9686	0.9923	0.9932		0.9638	
adjusted *R* ^2^			0.9607	0.9845	0.9864		0.9547	
predicted *R* ^2^			0.9299	0.9449	0.9545		0.9241	

a *; significant **; not significant, mean ± standard deviation. All analysis performed at least in triplicate.

**Figure 1:**
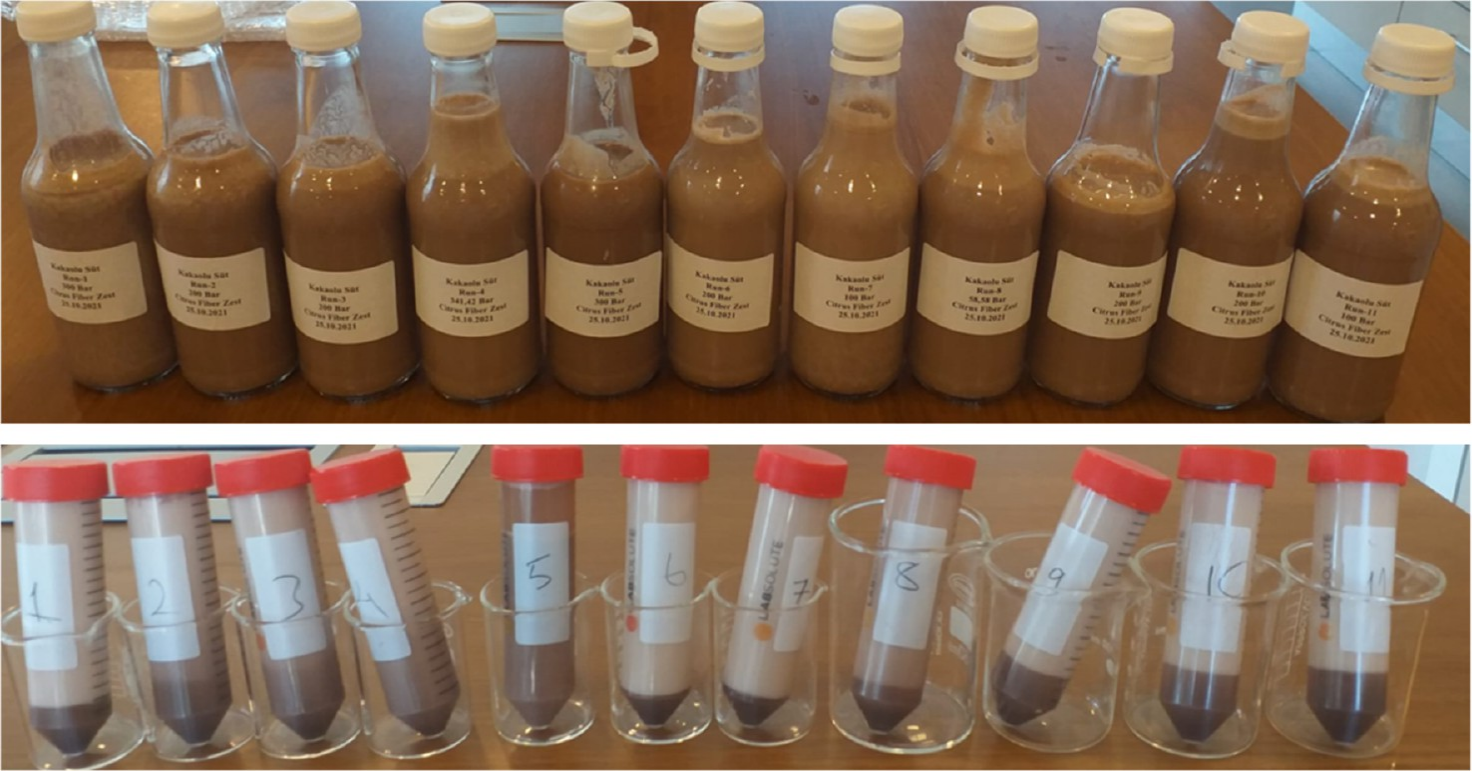
Images of milk-based cocoa beverages produced according to the CCRD experimental design.

### 3.1 Quality Properties of Milk-Based Cocoa Beverages

As shown in Figure 2, the WHC value increases significantly with the rise in citrus fiber. In addition, the increase in pressure also enhances the WHC value. The increase in homogenization pressure positively affected the WHC of the system, likely due to the mechanical disruption and dispersion of citrus fiber particles. Under higher pressure, the fiber’s physical structure becomes more open and accessible as the shear forces break down agglomerates and increase the surface area. This enhanced surface area allows the fiber matrix to better entrap and retain water, resulting in improved WHC. The intense mechanical shear and turbulence break down large fiber aggregates into finer particles. The WHC values vary between 75.65% and 97.60% (Table 1). The ANOVA test for WHC values was performed according to linear model conditions. According to this test, citrus fiber concentration, homogenization pressure, and their interaction were found to be statistically significant (*p* < 0.05), indicating that both the individual and combined effects of these parameters influenced the physical stability of the milk-based cocoa beverages. The *R* ^2^ value of the design is 0.9686, indicating the success of the model. In a study where the effect of the homogenization process on the WHC in an emulsion system consisting of water and oil using orange fiber was tested, it was found that the WHC value of the homogenized systems was approximately 30% higher than the WHC value of the nonhomogenized systems. It is seen that the homogenization process is mandatory if the WHC value is higher in systems where fibers are used or if it is desired to prevent or reduce syneresis. It is also thought that the homogenization process will provide a cost advantage for the producers. Since a more viscous product will be obtained when the homogenization process is applied, the desired texture can be obtained with less fiber use.^18^ The practical significance of increasing the WHC lies in its beneficial effects on the viscosity and stability of beverages. In milk-based cocoa beverages, higher WHC values contribute to maintaining homogeneity over extended periods, preventing phase separation. This enhancement prolongs shelf life, improves product stability, and delivers a more pleasant experience for the consumer. Beverages with a high WHC also reduce the separation of fat globules from the liquid, thereby improving physical properties and minimizing losses in freshness and flavor. Moreover, products with an elevated WHC offer a richer consistency and promote a greater sense of satiety for consumers.

**Figure 2:**
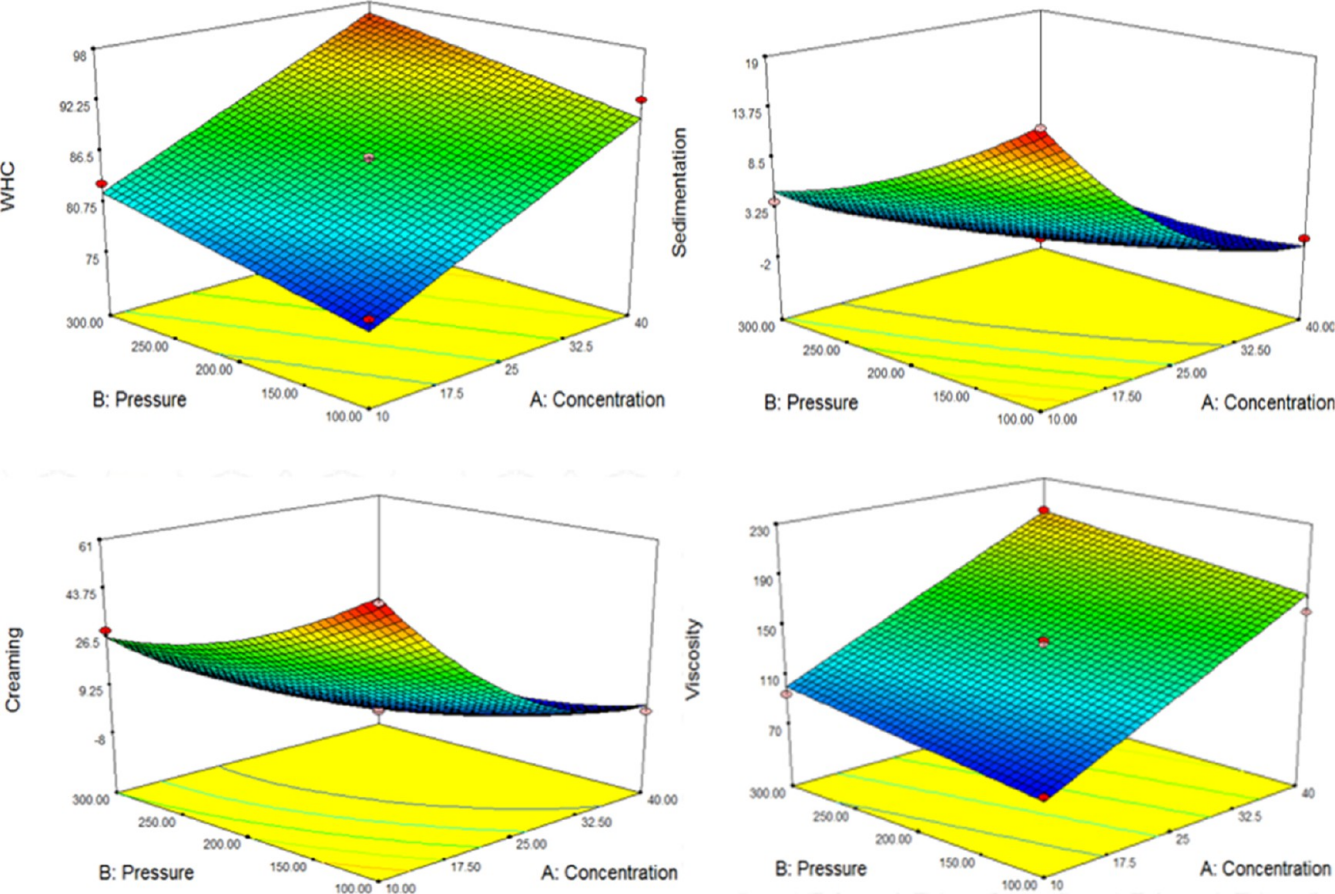
Effects of citrus fiber concentration and homogenization pressure parameters on dependent parameters of the samples.

According to the experimental design data, the sedimentation value (%) decreased as the citrus fiber ratio increased when the sedimentation value reached a certain level, and the subsequent increase did not make a difference. The increase in the pressure also affected the sedimentation value. Especially when increasing from 100 to 200 bar, the sedimentation value was significantly affected, and the increase after 200 bar did not affect it that much. As the amount of citrus fiber increases, the viscosity value also increases, so the probability of cocoa sedimentation decreases. The sedimentation value varies between 0% and 17.96% (Table 1). The quadratic model was suitable for explanation of the relation between independent variables and sedimentation with an *R* ^2^ value of 0.9923. As citrus fiber concentration had a statistically significant effect on the sedimentation value (*p* < 0.05), the homogenization pressure parameter was also found to be statistically significant (*p* < 0.05). The pressure and the amount of citrus fiber caused a statistically significant difference in the sedimentation value. When citrus fiber was 0.250% and above and the pressure value was 100 bar and above, the sedimentation value was found to be 0% (Figure 2). Here, the effect of both parameters on the sedimentation value is statistically significant (*p* < 0.05).

The creaming index value (%) decreases with the increase in citrus fiber up to a certain value. The increase in pressure increases the creaming index value statistically significantly. The creaming index value varies between 0% and 59.42% (Figure 2). The ANOVA test for creaming index values was performed according to quadratic conditions with a *R* ^2^ value of 0.9932, which is very close to 1 and indicates a strong correlation. According to this test, when citrus fiber concentration had a statistically significant effect on the creaming index value (*p* < 0.05), the homogenization pressure parameter was also found to be statistically significant on the creaming index value (*p* < 0.05). When citrus fiber is used at low concentration or when the pressure value drops below 100 bar, the creaming index value increases suddenly. It is thought that a stable system will be provided when the amount of fiber used and the applied homogenization process are optimized. Protein, pectin, and cellulose are the main components of citrus fiber. For this reason, it has been reported that fibers improve the ability to emulsify systems consisting of protein and polysaccharide and reduce the creaming index value.^3^

According to the applied experimental design data, viscosity values vary between 76.91 mPa·s and 225.80 mPa·s. For this reason, citrus fiber concentration created a statistically significant difference in the viscosity value. The ANOVA test for viscosity values was performed according to quadratic conditions. According to this test, the citrus fiber concentration had a statistically significant effect on both viscosity and homogenization pressure value (*p* < 0.05). The *R* ^2^ value of the design is 0.9638. As seen in Figure 2, increasing the citrus fiber ratio causes a statistically significant increase in the value of viscosity. The addition of citrus fiber leads to the formation of a more structured network in the liquid phase, contributing to higher viscosity. The torque value is proportional to the viscosity. The torque value also increases as the viscosity value increases. The torque value varies between 0.346 mN·m and 0.941 mN·m. The increase in the amount of citrus fiber in parallel with the viscosity value has caused the torque value to reach a higher value than the torque value of milk-based cocoa beverage samples produced with other hydrocolloids. In an emulsion system consisting of water and oil where citrus fibers were tested, the viscosity values of the emulsions formed before and after pressure application were measured. The study showed that the viscosity values of the products to which pressure was applied were higher. Mechanical processes applied to systems containing complex structures such as fibers ensure that the fiber bonds are opened and allow water to enter the bonds. This situation increases the WHC of the systems and prevents syneresis. At the same time, the viscosity of the system also increases.

### 3.2 Optimization

#### 3.2.1 Optimization Study

An optimization study was performed based on the results derived from the CCRD experimental design. The optimum point was determined to maximize the WHC value while minimizing sedimentation and creaming index values and ensuring pH, viscosity, and torque values within the range. The optimum point was determined as 0.400% citrus fiber concentration and 272.67 bar homogenization pressure. The desirability value of the optimization is 0.972. According to these conditions, the WHC value at the optimum point was found as 95.79%, sedimentation and creaming index value as 0%, and viscosity value as 196.90 mPa·s as predicted values. The predicted torque value was also given as 0.972 mN·m. At least three trials were conducted at the optimum point determined according to the model with the desirability function method (optimum process conditions), and the optimum point was also verified experimentally. The results of the verification trials conducted at the optimum point are shown in Table 2. Production was carried out according to the first optimization conditions selected. The results obtained because of optimization are quite close to the theoretical results and confirm these values.

**Table 2:** Optimum Point Obtained by CCRD Analysis Results of Optimum Point Sample^a^

products	WHC (%)	sedimentation (%)	creaming index (%)	pH	viscosity (mPa·s)	torque (mN·m)
optimum predicted	95.79	nd	nd	6.63	196.90	0.834
optimum experimental	95.95 ± 0.78	nd	nd	6.62 ± 0.02	197.22 ± 0.91	0.855 ± 0.027

a nd: not detected. The standard deviation is represented by ± values (*n* = 3). Different letters in each column (according to the Tukey test) are statistically significantly different (*p* < 0.05).

#### 3.2.2 Zeta Potential and Colloidal Stability

The zeta potential values of milk-based cocoa beverage samples formulated with citrus fiber averaged −27.90 mV (Table 3), indicating a moderately stable colloidal system. In colloidal dispersions, the zeta potential serves as a critical indicator of electrostatic stability. Systems with zeta potential values greater than ±25 mV are generally considered stable due to the repulsive forces that prevent aggregation or flocculation of particles.^19^ In this study, the relatively high absolute zeta potential, coupled with smaller particle sizes, suggests that the emulsions exhibited enhanced physical stability. Previous research has demonstrated that citrus fiber contains negatively charged polysaccharide structures that can adsorb to droplet interfaces, increasing surface charge and promoting electrostatic repulsion.^3^ These interactions reduce droplet aggregation and inhibit creaming by enhancing steric and electrostatic stabilization mechanisms. Thus, higher absolute zeta potential values correspond to stronger repulsive interactions between dispersed oil droplets, contributing to improved emulsion stability.^20^

**Table 3:** Analysis Results of Optimum Point Sample^a^

products	L*	a*	b*	*Z*-ave (nm)	zeta potential (mV)
optimum sample	50.37 ± 0.37	8.46 ± 0.16	9.62 ± 0.32	630.53 ± 25.39	–27.90 ± 0.29

a The standard deviation is represented by ± values (*n* = 3). Different letters in each column (according to the Tukey test) are statistically significantly different (*p* < 0.05).

#### 3.2.3 FTIR Spectroscopy

The FTIR spectra of milk-based cocoa beverage samples formulated with citrus fiber and without added hydrocolloids at the optimized formulation point are presented in Figure 3. A broad absorption band observed between 3100 and 3600 cm^–1^ is indicative of O–H stretching vibrations associated with hydroxyl groups, a characteristic feature of polysaccharides such as pectin and cellulose.^21^ This broadening is often attributed to intra- and intermolecular hydrogen bonding, suggesting the presence of hydrophilic groups that contribute to the water-binding capacity and stabilization of the beverage matrix. Additionally, a distinct absorption peak in the region of 1550–1650 cm^–1^ corresponds to the stretching vibrations of carbonyl (C=O) groups, which are commonly found in uronic acids of pectins and in amide or protein-related components potentially present in milk.^22^ This may also partially overlap with N–H bending vibrations from milk proteins, further complicating the spectral interpretation in this region. The peaks observed in the range of 1010–1080 cm^–1^ can be attributed to glycosidic linkages (C–O–C), representing the vibrational modes of C–O and C–C bonds in the polysaccharide backbone. This is consistent with previous findings related to starch and fiber-containing systems.^3,23^ These bands are characteristic of citrus-derived dietary fibers, such as cellulose, hemicellulose, and residual starches that may be present in citrus fiber preparations. Interestingly, although no new peaks emerged in the spectra of samples containing citrus fiber compared to those without added hydrocolloids, variations in the peak width and intensity were observed. These differences suggest molecular rearrangements or enhanced hydrogen bonding interactions resulting from fiber incorporation rather than the introduction of novel functional groups. Similar observations were reported, where the integration of polysaccharides into dairy systems altered the FTIR spectral profiles through changes in hydrogen bonding and network interactions rather than chemical composition.^7^ Thus, the FTIR analysis supports the hypothesis that citrus fiber, rich in polysaccharides and capable of extensive hydration and network formation, interacts physically rather than chemically with the milk beverage matrix, reinforcing stability without the need for synthetic hydrocolloids.

**Figure 3:**
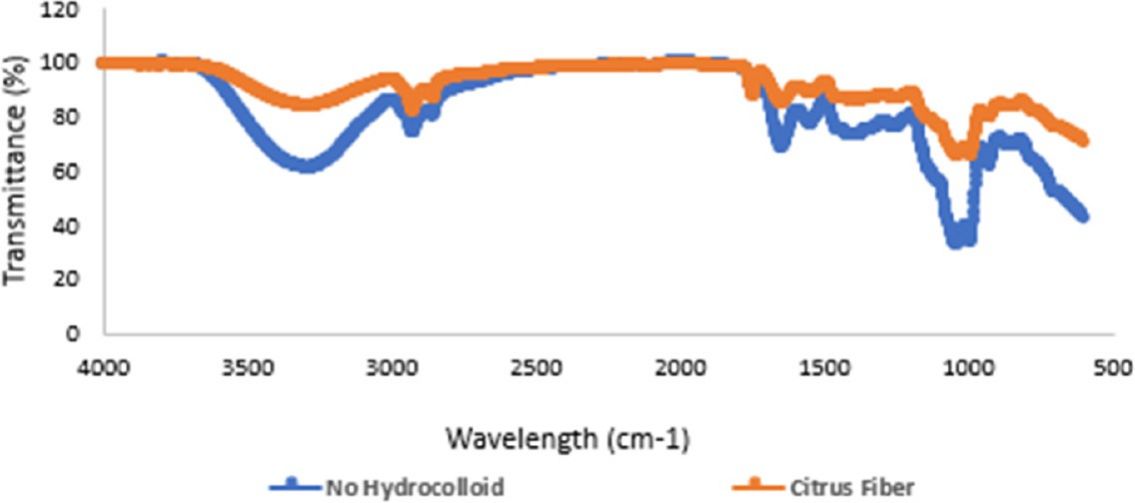
FTIR analysis results of no hydrocolloid and optimum point milk-based cocoa beverages.

#### 3.2.4 Microscopy Analysis

Microscopic evaluations at 10× and 40× magnifications of milk-based cocoa beverage samples with citrus fiber at the optimized formulation point and the other representing a leading commercial product from the local market are presented in Figure 4. Notably, the sample formulated with citrus fiber exhibited a significantly higher viscosity and a more stable dispersion of cocoa particles compared to the commercial counterpart. This observation aligns with prior research suggesting that citrus fiber, rich in soluble and insoluble polysaccharides, enhances emulsion and suspension stability by increasing viscosity and forming a three-dimensional network that entraps dispersed particles.^3^ To evaluate the microstructural stability, localized image analysis was conducted by extracting nonoverlapping window segments (*W* × *W* patches) and calculating textural descriptors, including entropy and variance. The sample containing citrus fiber consistently exhibited lower entropy and variance values across various window sizes, indicating a more uniform and organized internal structure. As entropy reflects the degree of randomness or disorder in pixel intensity distribution, lower entropy suggests better spatial consistency, correlating with reduced particle migration or aggregation.^24^ Similarly, lower variance values in the citrus-fiber-containing sample confirm less deviation in pixel intensity, indicating a smoother and more cohesive dispersion of cocoa particles throughout the matrix. These findings align with previous microscopy-based studies in emulsion and dispersion systems, where lower entropy and variance correlated with better ingredient distribution and physical stability.^25,26^ Furthermore, Gray Level Co-occurrence Matrix (GLCM) features, particularly the homogeneity metric, which quantifies the similarity of neighboring pixel values, were also used to assess spatial uniformity. The optimized sample achieved higher homogeneity values, indicating stronger pixel intensity correlations and thus a more consistent and stable structure. This further supports the interpretation that citrus fiber promotes uniform distribution of cocoa solids, likely due to its ability to bind water and create a steric barrier against particle sedimentation.^7^ Collectively, these microscopy findings validate the stabilizing effect of citrus fiber in milk-based cocoa beverages, offering a clear functional advantage over conventional commercial formulations lacking such targeted structural support.

**Figure 4:**
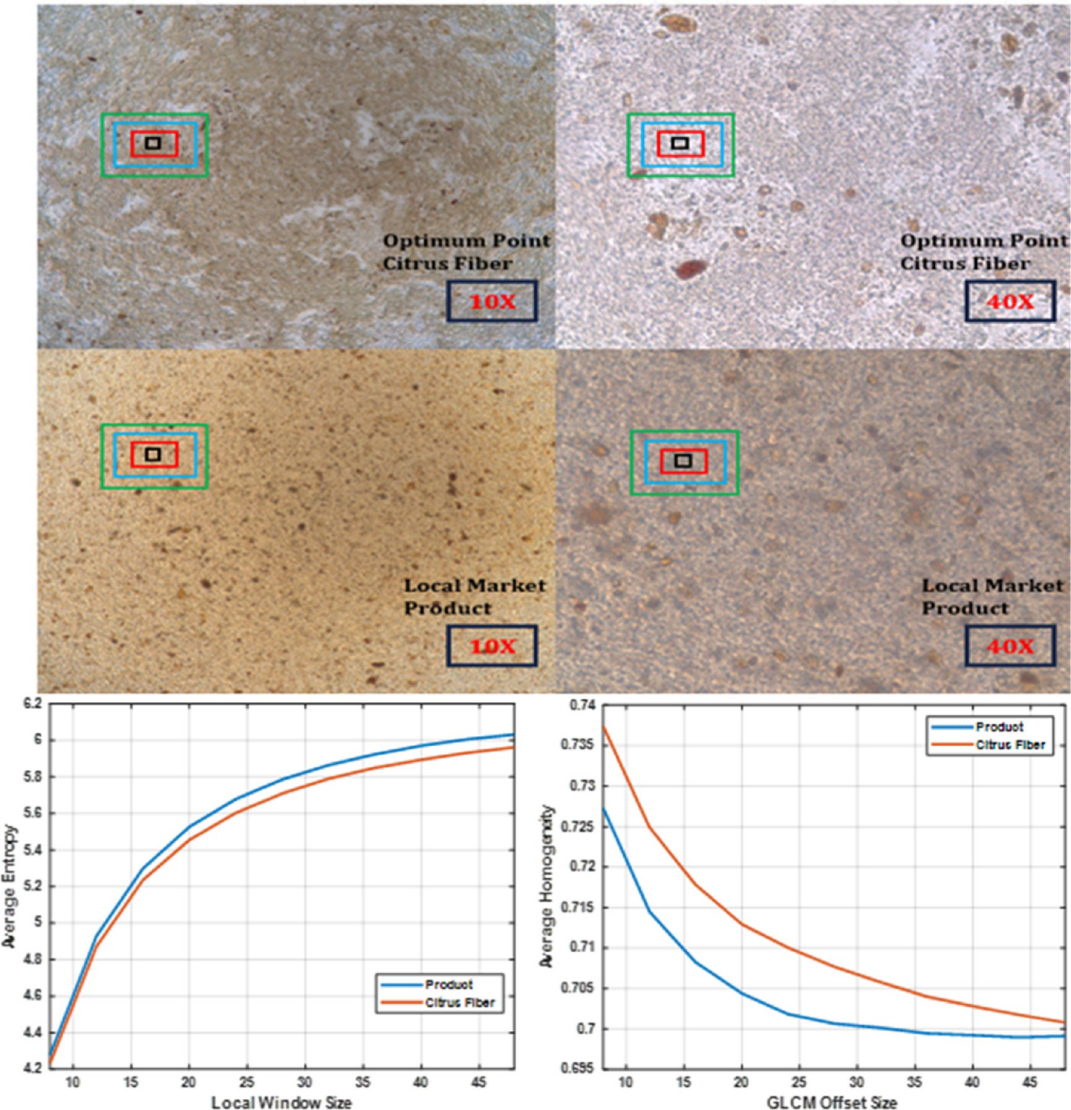
Microscopic images of optimum point milk-based cocoa beverages and local market product at 10- and 40-times magnification with metrics extracted from nonoverlapping patches.

#### 3.2.5 Scanning Electron Microscopy (SEM)

SEM images of milk-based cocoa beverage samples produced with citrus fiber at the optimum point are shown in Figure 5. No air gaps were observed in the SEM images of milk-based cocoa beverage samples produced with citrus fiber at the optimum point after drying in a freeze-dryer. When compared to the images without a hydrocolloid, a more holistic structure was observed. An additional analysis was performed using Scanning Electron Microscopy (SEM) images to examine the structural characteristics of three samples including no hydrocolloid, optimum point, and local market samples. The findings highlighted the remarkable stability of the optimum point, which demonstrated the lowest entropy and variance values, signifying a highly compact and well-organized network. This can be attributed to both the selected ingredients and the controlled processing conditions, which play critical roles in microstructural formation. In the formulated sample, the use of citrus fiber, along with a precise homogenization pressure, contributes to a tightly bound network with efficient particle dispersion. Citrus fiber introduces a structured plant cell wall matrix capable of forming hydrogen bonds and interacting with proteins and fat droplets, leading to enhanced stabilization and reduced microstructural randomness (entropy). Moreover, the homogenization step at controlled pressure levels (as per the CCRD design) contributed to a uniform particle size distribution, promoting consistent dispersion and reducing the likelihood of phase separation. This effect is indirectly supported by the stability results observed during storage. Specifically, almost no creaming was detected during 9 months of storage in milk-based cocoa beverages stabilized with 0.400% (w/w) citrus fiber, suggesting that the droplet size distribution remained stable over time. Flocculation and coalescence are phenomena typically associated with an evolving droplet size distribution. Creaming is often a precursor to both of these processes.^27^ The absence of creaming provides strong evidence of the physical stability of the emulsion system. Long-term creaming resistance supports the effectiveness of both the homogenization process and citrus fiber as a stabilizing agent. The controlled heat treatment and rapid cooling further prevent protein denaturation or aggregation, supporting network integrity. In contrast, the no hydrocolloid and local market sample products often rely on less targeted stabilization strategies. The absence of functional hydrocolloids or the use of generic stabilizers may result in heterogeneous droplet sizes and poor interaction between dispersed and continuous phases, resulting in higher entropy and variance. Additionally, commercial products may undergo ultrahigh temperature (UHT) processing and extended storage, which can alter protein structures and destabilize emulsions if not optimized. Thus, the lower entropy in the formulated beverage arises from both the synergistic effect of tailored ingredient selection and optimized process parameter factors that are often not as precisely controlled in commercially available beverages. Moreover, the optimum point exhibited the highest homogeneity values, reflecting its consistent and tightly organized system, likely due to the stabilizing role of citrus fiber. These observations are consistent with the results obtained from microscopy image analyses, reinforcing the conclusion that the optimum point sample offers superior structural stability.

**Figure 5:**
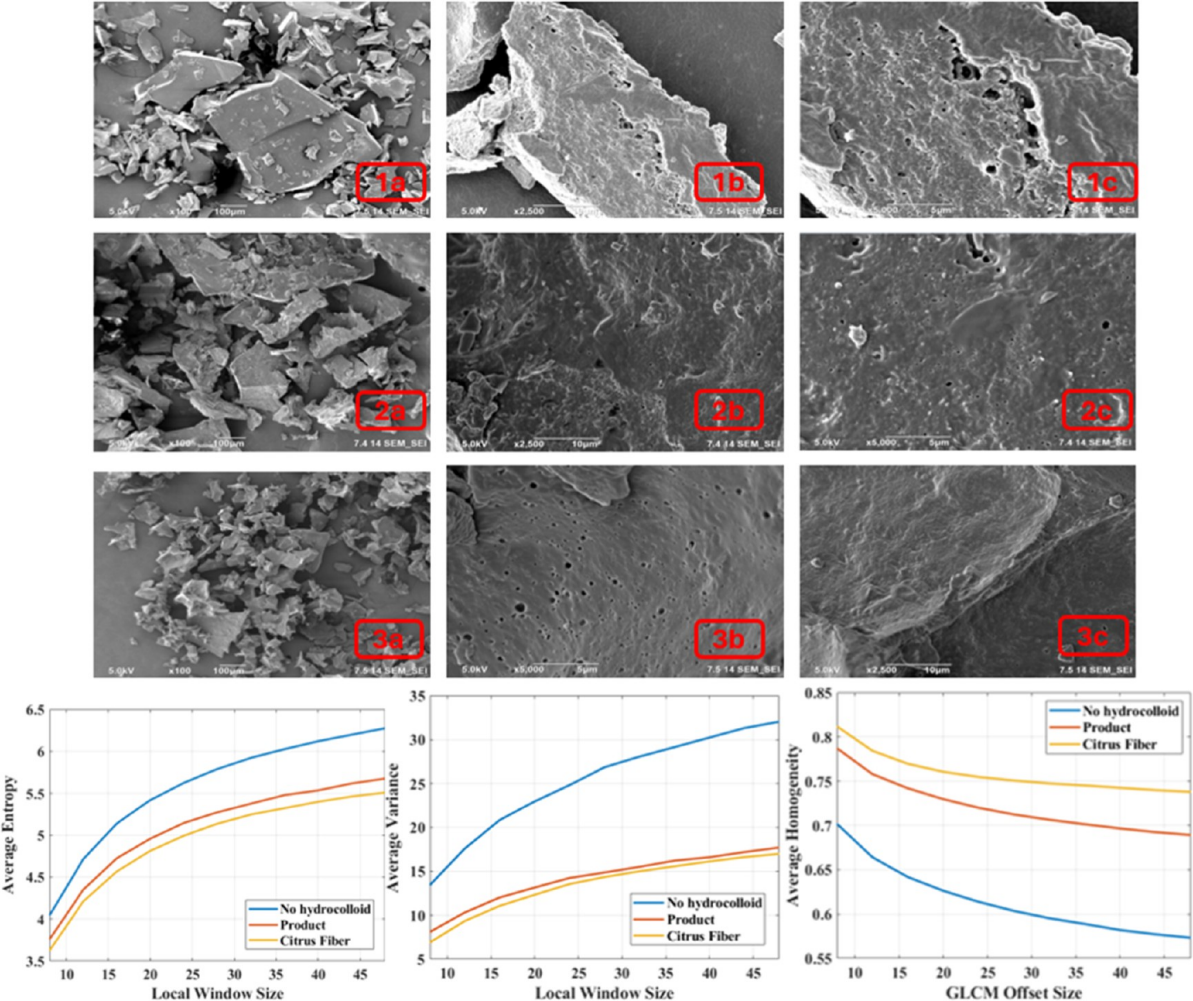
SEM images of milk-based cocoa beverages without hydrocolloid (100, 2500, and 5000 magnification (1a, 1b, and 1c, respectively), optimum point (100, 2500, and 5000 magnification (2a, 2b, and 2c, respectively), and local market product (100, 2500, and 5000 magnification (3a, 3b, and 3c, respectively) with metrics extracted from nonoverlapping patches.

#### 3.2.6 Elemental Composition (EDS)

Figure 6 and Table 4 present the elemental composition analysis of milk-based cocoa beverage samples, comparing a formulation containing citrus fiber at the optimum point with a control sample without hydrocolloids. EDS results revealed that the beverage containing citrus fiber exhibited elevated levels of both phosphorus and potassium compared with the hydrocolloid-free counterpart. This increase in phosphorus and potassium concentrations can be attributed to the functional interactions between citrus fiber and the milk matrix. Citrus fiber, rich in pectic substances and organic acids (such as galacturonic acid), tends to lower the system’s pH due to its intrinsic acidity.^28^ In milk-based systems, such a drop in the pH can shift the mineral equilibrium, potentially destabilizing casein micelles or altering salt balances. To restore pH and ionic strength, buffering agents such as potassium phosphate are often introduced during processing, resulting in increased incorporation of K^+^ and PO_4_^3–^ ions, which are subsequently detected via EDS.^29^ This explanation aligns with prior findings showing that low-pH polysaccharide additions require mineral buffering to stabilize protein systems in dairy matrices.^30^ Moreover, previous research confirms that pectin and dietary fiber components can bind and interact with mineral ions, influencing their retention and distribution in complex food systems.^31,32^

**Figure 6:**
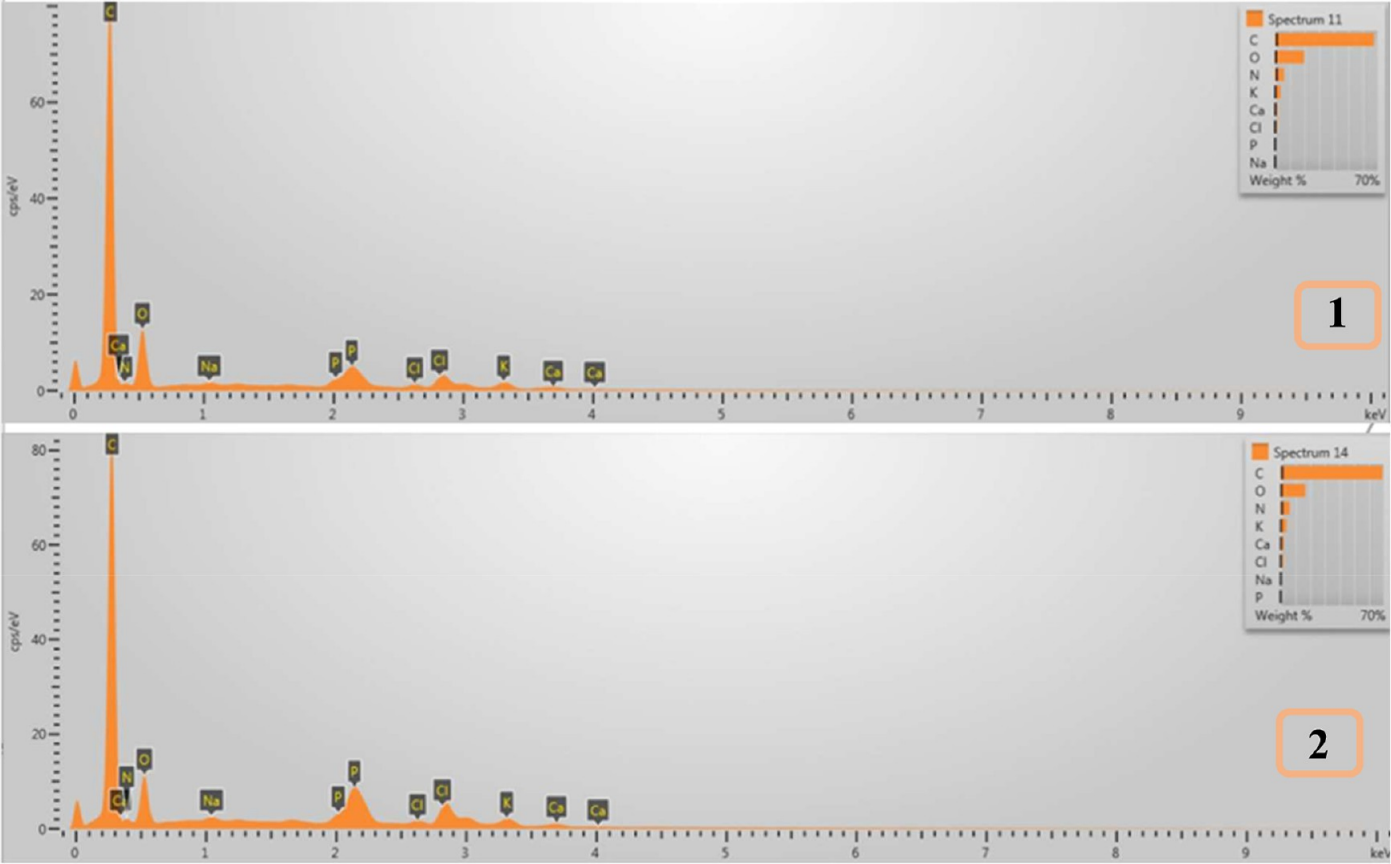
EDS images of milk-based cocoa beverages without hydrocolloid (1) and optimum point (2) (elements written on the peaks are C, Ca, N, O, Na, P, P, Cl, Cl, K, Ca, and Ca, respectively).

**Table 4:** EDS Results of Milk-Based Cocoa Beverages without Hydrocolloid and Optimum Point Sample^a^

products	C (%)	N (%)	O (%)	Na (%)	P (%)	Cl (%)	K (%)	Ca (%)
no hydrocolloid	69.30 ± 0.15^a^	6.63 ± 0.83^a^	16.63 ± 0.18^b^	0.56 ± 0.06^a^	0.00^b^	1.38 ± 0.11^a^	3.79 ± 0.10	1.72 ± 0.38^a^
optimum sample	64.75 ± 3.89	5.77 ± 0.23	22.71 ± 2.78^a^	0.43 ± 0.10	0.40 ± 0.06^a^	1.33 ± 0.21	4.15 ± 0.80^a^	1.00 ± 0.11^b^

a The standard deviation is represented by ± values (*n* = 3). Different letters in each column (according to the Tukey test) are statistically significantly different (*p* < 0.05).

The elevated potassium and phosphorus levels seen in EDS analysis therefore reflect not only the compositional influence of citrus fiber itself but also the formulation strategies employed to maintain product stability and sensory quality. These findings provide further evidence of citrus fiber’s multifaceted role, not only as a stabilizer and thickening agent but also as a component that necessitates specific mineral adjustments in product development.

### 3.3 Molecular Docking

The interactions of citrus fiber with α-casein, β-casein, α-lactalbumin, and β-lactoglobulin milk proteins were investigated by molecular docking analysis, Figure 7. Since the main component of citrus fiber is cellulose (40%), the interactions of cellulose as ligand and milk proteins were analyzed. Free binding energies ranged from −4.8 kcal/mol to −6.4 kcal/mol. Interactions between β-lactoglobulin and cellulose showed the strongest binding affinity with a binding energy of −6.4 kcal/mol, while the binding between α-casein and cellulose was the weakest and calculated as −4.8 kcal/mol. Docking analyses revealed two hydrogen bonds and a salt bridge in the interactions between α-casein and cellulose. Hydrogen bonds occurred between the amino acid residues Phe38 and Arg166 and cellulose, and hydrogen bonds were detected at distances of 2.87 Å with Phe38 and 2.38 Å with Arg166. In addition, a salt bridge was formed between Arg166 and cellulose at a distance of 4.42 Å and this bond was stabilized by a carboxylate group. The interactions between β-casein and cellulose involve a total of eight hydrogen bonds. These hydrogen bonds were formed between serine (Ser383), threonine (Thr388), aspartic acid (Asp454), and glutamic acid (Glu455) residues and cellulose. Hydrogen bonds were detected at distances of 2.08 Å with Ser383, 2.48 Å and 2.16 Å with Thr388, 3.09 Å and 3.26 Å with Asp454, and 2.51 Å and 3.26 Å with Glu455. The analyses reveal that these bonds are mostly formed between the hydroxyl groups of cellulose and the side chains of the protein residues. The binding energy was calculated as −6.0 kcal/mol, indicating that strong hydrogen bonds play a critical role in the stability of the β-casein and cellulose complex. Four hydrogen bonds were detected in the interactions between α-lactalbumin and cellulose. These hydrogen bonds occurred between histidine (His32), glutamic acid (Glu49), leucine (Leu105), and alanine (Ala106) residues and cellulose. Hydrogen bonds were detected at distances of 2.20 Å with His32, 2.29 Å with Glu49, 2.67 Å with Leu105, and 2.23 Å with Ala106. These bonds are considered to stabilize interactions between proteins and cellulose. The binding energy was calculated as −5.4 kcal/mol. A total of six hydrogen bonds were found in the interactions between β-lactoglobulin and cellulose. Hydrogen bonds occurred between cellulose and amino acid residues such as tyrosine (Tyr20, Tyr42), glutamic acid (Glu45), and glutamine (Gln59). Hydrogen bonds were detected at distances of 2.80 Å with Tyr20, 2.26 Å with Tyr42, 2.08 Å and 2.10 Å with Glu45, and 2.67 Å and 2.04 Å with Gln59. The binding energy was calculated as −6.4 kcal/mol, indicating the formation of a strong complex between β-lactoglobulin and cellulose. These findings align with experimental results indicating that homogenization pressure and citrus fiber concentration interact synergistically to enhance specific physical stability parameters of the beverage. Notably, this synergy was reflected in the zeta potential value (−27.90 mV), which indicates increased electrostatic repulsion between particles and reduced aggregation. The combined effect of high homogenization pressure (which reduces particle size and enhances dispersion) and citrus fiber (which interacts with milk proteins) appears to optimize the surface charge distribution, thereby stabilizing the colloidal network. This molecular-level interaction likely contributes to the observed improvements in sedimentation stability, water-holding capacity, and creaming behavior.

**Figure 7:**
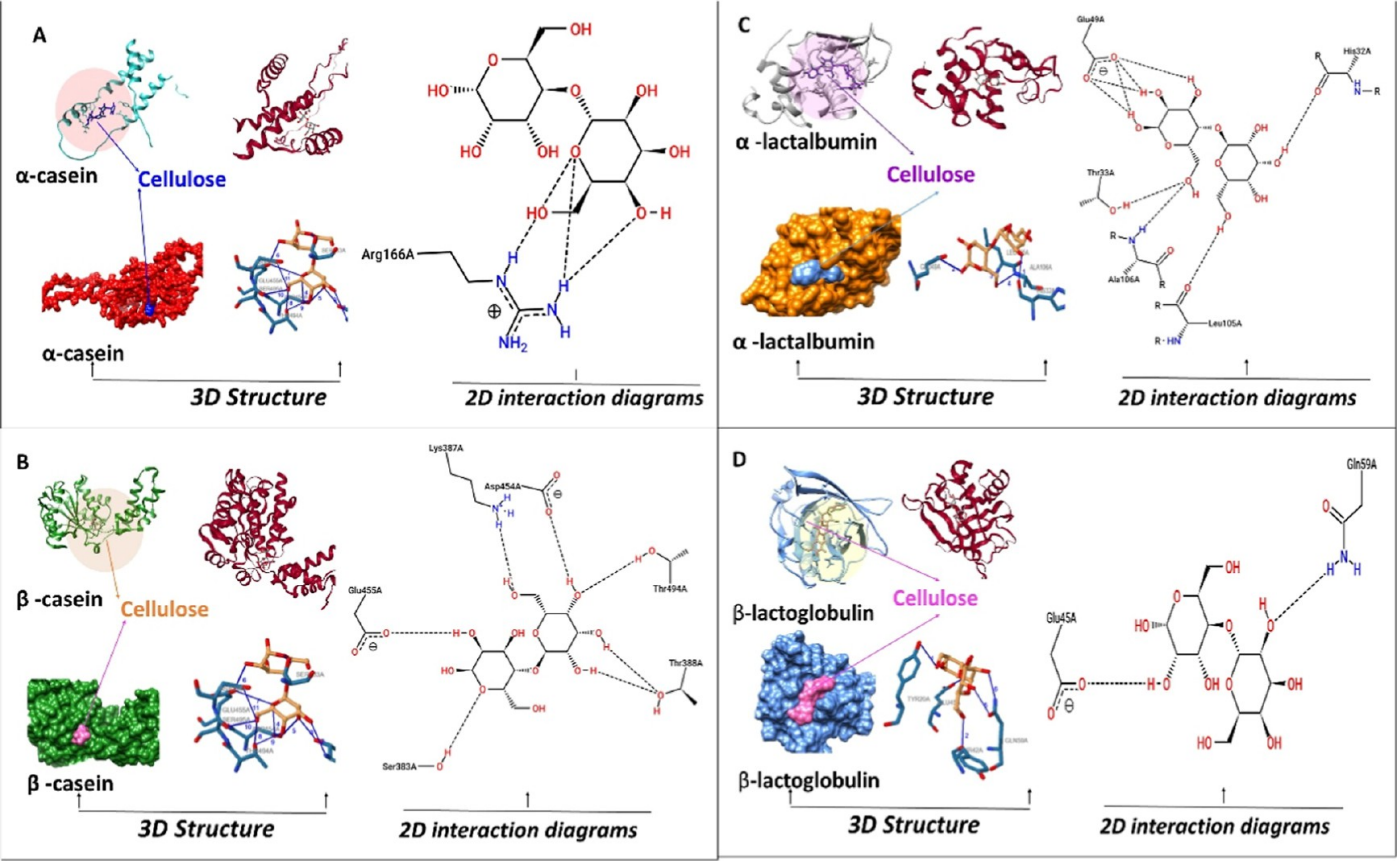
Molecular docking surface 3D structures and 2D interaction diagrams of α-casein (A), β-casein (B), α-lactalbumin (C), and β-lactoglobulin (D), respectively.

### 3.4 Sensory Evaluation

Figure 8 presents the sensory evaluation scores for milk-based cocoa beverages formulated with citrus fiber at the optimum point versus those prepared without hydrocolloids. Among the evaluated attributes, smoothness emerged as a critical parameter defined by the absence of gritty or rough textures upon oral contact. Smoothness is particularly important in emulsion-based beverages, as it indicates efficient dispersion of cocoa solids and the absence of large or aggregated particles.^33^

**Figure 8:**
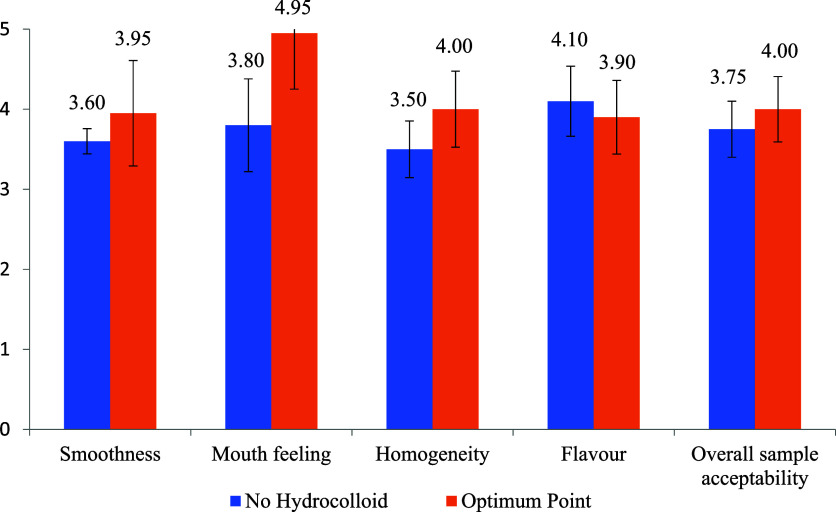
Sensory evaluation scores for no hydrocolloid and optimum point milk-based cocoa samples.

Panelists consistently favored the citrus fiber-stabilized samples, citing a superior mouthfeel, characterized by enhanced viscosity, creaminess, and an even coating on the palate. These desirable qualities align with prior studies, suggesting that dietary fibers such as citrus fiber improve emulsion stability and increase the perceived creaminess of beverages by forming stable aqueous networks. In contrast, the hydrocolloid-free samples were reported to exhibit a rougher mouthfeel, likely due to sedimentation or insufficient particle suspension, a common limitation in unstabilized cocoa milk formulations.^34,35^

Mouthfeel, encompassing viscosity, richness, and creaminess, was perceived as more pleasant in the optimized samples. The use of citrus fiber likely contributed to these improvements through its water-holding capacity and ability to form a gel-like matrix, which provides a lubricating effect during consumption.^36^

Homogeneity was another distinguishing factor. A uniform distribution of particles ensures consistent color, flavor, and texture, all of which influence consumer acceptability. The optimized beverage demonstrated greater homogeneity than its counterpart without hydrocolloids. This is consistent with findings from hydrocolloid-based food systems, where well-structured networks minimize sedimentation and improve sensory uniformity.^37^

Regarding flavor, panelists noted that while the optimized sample maintained acceptable cocoa notes, it also exhibited slight citrus undertones. These were attributed to the intrinsic aromatic compounds of the citrus fiber. Interestingly, the beverage without hydrocolloids was rated slightly higher in cocoa intensity, possibly due to the absence of flavor-masking or competing aromatic compounds. Nevertheless, no off flavors, such as bitterness or sourness, were reported in either formulation, indicating that the citrus fiber did not detract significantly from the overall flavor quality.

The overall acceptability score was higher for the optimized formulation. This reflects not only its improved texture and homogeneity but also the balanced sensory profile that resulted from the stabilization effect of citrus fiber. These findings support previous research demonstrating that well-structured and stable emulsions enhance consumer satisfaction in dairy-based beverages.^38^

### 3.5 Storage Analysis

Table 5 shows the changes in the quality parameters of the milk-based cocoa beverage produced under optimum conditions after 9 months of storage. The WHC, sedimentation, creaming index, pH, viscosity, and torque values of the optimum point samples were examined every month. The long-term stability of milk-based cocoa beverages formulated with citrus fiber was evaluated over a 9 month storage period. WHC remained statistically unchanged for the first 7 months (*p* > 0.05), with a significant change detected in the eighth month (*p* < 0.05), after which no further statistically significant variation was observed throughout the ninth month. This stability highlights the water-binding efficiency of citrus fiber, which is known to improve the WHC of emulsified food systems due to its high content of insoluble polysaccharides and its ability to form a three-dimensional network structure.^39^ Most notably, sedimentation of cocoa particles was completely absent throughout the 9 month shelf life, as indicated by a sedimentation value of 0. In contrast, commercial market samples exhibited clear signs of cocoa particle sedimentation, supporting the prediction that citrus fiber can serve as an effective clean-label-friendly stabilizer. Similar findings have been reported, which demonstrated that dietary fibers, particularly from citrus sources, form particulate networks that enhance physical stability in emulsion systems.^3^ Additionally, creaming index values at the optimum formulation point remained statistically unchanged during the entire storage period (*p* > 0.05). This indicates minimal phase separation, a key attribute of emulsion stability. These results support the emulsification capacity of citrus fiber, which likely stabilizes oil-in-water interfaces via both steric and electrostatic mechanisms.^3^ Visual and structural evidence from SEM and light microscopy (Figures 4 and 5) further confirmed the homogeneity and microstructural integrity of the system. In terms of pH stability, no significant changes were observed (*p* > 0.05), reinforcing the buffering effect and formulation robustness of citrus fiber-enriched systems. Meanwhile, viscosity values showed a statistically significant increase beginning from the third month (*p* < 0.05), and torque values also rose significantly from the sixth month onward. This progressive thickening may result from the further hydration or cross-linking of fiber structures over time, a phenomenon previously observed in storage studies of fiber-stabilized emulsions.^40^ Collectively, these findings suggest that citrus fiber contributes to long-term physical stability by enhancing viscosity and WHC, preventing sedimentation and creaming, maintaining microstructural homogeneity, preserving emulsion pH, and possibly stabilizing zeta potential, which minimizes particle aggregation and phase separation.^3^ Such attributes position citrus fiber as a clean-label-friendly stabilizer that can replace synthetic hydrocolloids, meeting both consumer demand for recognizable ingredients and industrial need for reliable shelf life performance.

**Table 5:** 9 Months Storage Results of Optimum Point Sample^a^

months	WHC (%)	sedimentation (%)	creaming index (%)	pH	viscosity (mPa·s)	torque (mN·m)
1	95.80 ± 0.42^a^	nd	nd	6.63 ± 0.02^a^	204.95 ± 6.29^a^	0.857 ± 0.010^a^
2	95.45 ± 0.49^a^	nd	nd	6.61 ± 0.01^a^	213.45 ± 3.46^a^	0.896 ± 0.005^a^
3	94.80 ± 0.14^a^	nd	nd	6.60 ± 0.03^a^	225.90 ± 4.53^b^	0.941 ± 0.007^a^
4	93.75 ± 1.06^a^	nd	nd	6.58 ± 0.02^a^	238.43 ± 3.80^bc^	0.996 ± 0.006^a^
5	93.18 ± 0.32^a^	nd	0.01 ± 0.0008^a^	6.58 ± 0.04^a^	244.38 ± 4.61^c^	1.020 ± 0.007^a^
6	92.85 ± 0.21^a^	nd	0.01 ± 0.0011^a^	6.56 ± 0.01^a^	259.70 ± 3.11^cd^	1.084 ± 0.004^b^
7	91.03 ± 0.32^a^	nd	0.02 ± 0.0016^a^	6.53 ± 0.04^a^	276.69 ± 3.44^d^	1.153 ± 0.011^b^
8	90.30 ± 0.14^b^	nd	0.02 ± 0.0021^a^	6.51 ± 0.03^a^	290.08 ± 2.52^e^	1.212 ± 0.009^b^
9	89.65 ± 0.35^b^	nd	0.03 ± 0.0016^a^	6.49 ± 0.05^a^	305.10 ± 6.08^e^	1.278 ± 0.012^b^

a nd: not detected. The standard deviation is represented by ± values (*n* = 3). Different letters in each column (according to the Tukey test) are statistically significantly different (*p* < 0.05).

## 4 Conclusion

This study demonstrates that incorporating clean-label-friendly citrus fiber into milk-based cocoa beverages effectively improves their physicochemical and structural stability. The optimized formulation exhibited enhanced water-holding capacity and resistance to sedimentation and creaming and maintained consistent pH and microstructural integrity over a 9 month shelf life. The stable zeta potential and molecular size distribution further contributed to a homogeneous and shelf-stable emulsion. These results suggest that citrus fiber not only meets clean-label demands but also offers functional benefits, making it a promising alternative to synthetic stabilizers in dairy-based beverages.

Future investigations should explore consumer sensory perception in diverse demographic groups and evaluate process scalability and cost-efficiency for industrial implementation. Overall, citrus fiber presents a viable strategy for developing high-quality, stable, and market-ready cocoa beverages aligned with consumer preferences and clean-label trends.
